# Do rainfalls wash out anthropogenic airborne magnetic particulates?

**DOI:** 10.1007/s11356-017-8638-9

**Published:** 2017-03-01

**Authors:** Amarjargal Baatar, Raegyung Ha, Yongjae Yu

**Affiliations:** Department of Astronomy, Space Science, and Geology, Chungnam National University, Daejeon, 34134 South Korea

**Keywords:** Rainfalls, Asian dust storms, Anthropogenic pollutions, Precipitation, Particle size, Monthly variation, Magnetic material, Heavy metals

## Abstract

We separated dust particles from the mesh-filtered sets of rainwaters collected on rainy days with daily precipitations exceeding 10 mm per day. A total of 136 rainwaters (or snow during the winter season) samples collected from February 2009 to February 2013 were analyzed. In particular, 33 out of 136 rainwaters were collected during or just after the Asian dust storm (ADS) events. Values of pH were relatively higher during warmer seasons. During ADS events, precipitations were alkaline, possibly due to abundant supply of alkaline minerals from the deserts source area to the precipitation. Compositional analysis on particulate matter (PM) indicated that Fe (and Al, K, and Mg) enriched the dusts collected during ADS, with respect to events than those without ADS. We found that ADS rainfall events are effective in selectively eliminating dust particles. However, high rainfall does not necessarily indicate more dilution of dusts. On microscopic examination, we observed natural soils, natural dust of pedogenesis or weathering origin, anthropogenic C–Fe-rich particles, and anthropogenic C-rich particles. Because of its small size, the stoichiometry of ADS-related, Fe-rich dust particles was inferred from the magnetic analysis. Presence of Verwey transition near 100–120 K and experimental determination of Curie points near 580 °C indicate that magnetic mineral responsible for the magnetic properties of ADS-related dusts was magnetite.

## Introduction

Air pollution has acute impact on human health as the average adult, at rest, consumes over 10^4^ L of air in a day (Koenig 2012). Urban air pollution results both from anthropogenic and natural sources, although it is mostly caused by human activities. Anthropogenic air pollution involves emission of harmful material including black carbon, heavy metals, and sulfates into atmosphere, causing disease or even death of living organisms (Guo et al. 2004; Hsu et al. 2004; Ramana et al. 2010). In practice, particulate matter (PM) is the most hazardous pollution component widely present in the environment (Donaldson 2003). It is true that natural events including evaporation of organic material, forest fires, pollen disposal, and volcanic outgassing also contribute to the air pollution.

Asian dust storm (ADS) is often reworded as yellow dust, yellow sand, yellow wind, or China dust storms in the literature. It is produced when high-speed surface winds soar dense clouds of solid particles from the Gobi and Taklamakan deserts. Such uprising airflow generates buoyant energy to form ADS. Prevailing westerly winds spread the ADS along the stratosphere or upper troposphere. Once produced, small PM (particulates smaller than 5 μm in diameters (PM_5_)) can be transported across East Asia, and the Pacific, as far away as North America (Ma et al. 2001).

ADS has effects on the global climate system by affecting the radiation budget, atmospheric chemistry, and the air quality and human health (Kim et al. 2003; Ramana et al. 2010). In addition, the worst air quality during seasonal ADS events is notorious as it is directly relevant to the cardiovascular and respiratory diseases (Kwon et al. 2002). Anthropogenic pollution is probably intensified as the winds pass over eastern China, which is currently the world largest sulfur dioxide emitter (e.g., Shu et al. 2000, 2001; Wang et al. 2007, 2008). In addition, increase of desertification in Asia produced ADS more frequently over the last few decades.

Quantitative estimates of past rainfall and magnetic tracing of PM are now available from high-resolution magnetic measurements on dust particles collected from ancient soils or rocks (e.g., Maher et al. 1994; Maher 2009; Liu et al. 2012). In particular, the high-resolution sensitivity, rapid measurement, and non-destructive nature of magnetic measurements make magnetic monitoring as a useful tool for detecting environmental signals associated with anthropogenic PM (e.g., Salome and Meynadier 2004; Chaparro et al. 2006; Sagnotti et al. 2006). Over the last few decades, physicochemical influence of dust particulates on rain has been explored (e.g., Ro et al. 2001; Avila et al. 1998; Seto and Hara 2006). The aim of present study is to check the wash-out effects of rainfalls on anthropogenic PM in South Korea, by relating precipitation chemistry of rainfalls and magnetic properties of PM with or without ADS.

## Materials and methods

**Fig. 1:**
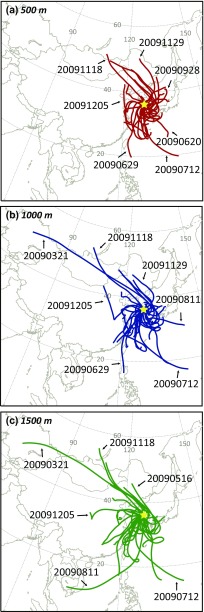
Location map of study area with examples of backward trajectory using Hybrid Single-Particle Lagrangian Integrated Trajectory (HYSPLIT). Trajectories at **a** 500 m, **b** 1000 m, and **c** 1500 m above mean sea level (amsl) were traced for 72 h for 33 ADS events

Korea is located in a downstream bottleneck of prevailing westerly winds in East Asia. The study area, Daejeon metropolitan city, is the fifth largest city of South Korea with a population over 1.5 million, located about 20 km away from the Sejong City (a new administrative capital of South Korea). The wet sample collector was installed on the roof of five-story building W11–1, Chungnam National University (36° 21′ 58.82″ N, 127° 20′ 23.95″ E) (Fig. 1).

Temporal variations of dust density in air and the amount of rainfall were officially recorded in local stations operated by Korea Meteorological Administration (KMA). A total of 136 rainwaters (or snow during the winter season) samples collected from February 2009 to February 2013 were analyzed in this study. In particular, 33 out of 136 rainwater samples were collected during or just after the ADS events, with a hope to directly compare the properties of PM before and after the ADS events. Path of air parcel can be traced using the Hybrid Single-Particle Lagrangian Integrated Trajectory (HYSPLIT) model (Draxler and Hess 1998) associated with global data assimilation system (Stein et al. 2015). The HYSPLIT model (http://ready.arl.noaa.gov/HYSPLIT.php) for 33 ADS events were displayed for 72 h at altitudes of 500 m (Fig. 1a), 1000 m (Fig. 1b), and 1500 m (Fig. 1c).

For each rainwater sample, more than 2000 mL of rainwater was initially collected. On rainwater collection, values of pH and electrical conductivity (EC) were measured. For each pH measurement, the pH electrode was calibrated three times, using buffer solutions with pH of 4.00, 7.00, and 10.00, respectively. To extract solid particles from the rainfall, rainwater samples were filtered successively with six different sequentially down-sizing membrane filters with pore sizes of 5.0, 3.0, 1.0, 0.45, 0.2, and 0.1 μm.

Morphology and chemical composition of the dust particles extracted have been analyzed by using scanning electron microscope (SEM) (FE-SEM JSM-7000F) and electron probe X-ray microanalyzer (model JXA8800R). At first, solid particles present in membrane filters were analyzed by SEM with energy-dispersive X-ray spectroscope (EDS). On completion of SEM and EDS analyses, particles were selected in largest order for further compositional analysis for each rain-filtered PM_5_. A total of 1030 (103 samples × 10 grains per sample) and 990 (33 samples × 30 grains per sample) compositional determinations were carried out using electron probe X-ray microanalyzer (EPMA) for solid extracts from non-ADS and ADS events, respectively. The operating conditions of SEM used an accelerating voltage of 15 keV with a sample current of 5.0 nA for energy-dispersive analysis. For quantitative EPMA analysis, we set signal collecting times for 100 s and background countings for 50 s. On the basis of previous experience, we now know that dust particles often contain carbon-bearing materials (e.g., Kim et al. 2007, 2008, 2009). Hence, platinum coating rather than carbon coating was applied prior to compositional analysis.

Magnetic analysis was focused on characterization of solid particles extracted from 33 rainfall events during or just after the ADS events. For magnetic analysis, solid particles of PM_5_ collected from fiber filters were tightly wrapped in a non-magnetic plastic straw. Dust particles extracted from rainfalls were subjected to stepwise isothermal remanent magnetization (IRM) acquisition up to a field of 1 T. The peak fields were applied with an ASC Scientific IM-10 impulse magnetizer. All the magnetic experiments were measured on a JR6 spinner magnetometer (AGICO, Brno, with a noise level of 10^−11^ A m^2^). We secured enough dusts from 33 ADS-related rainfall events that are measurable on magnetic instruments with magnetic moment at least an order stronger than the noise level. Unfortunately, dusts collected from 103 non-ADS-related raining events were magnetically too weak with magnetic moment similar to or even less than the noise level. As a quick and non-destructive technique, IRM is dependent on the magnetic mineralogy, concentration, and distribution of magnetic coercivity. IRM component analysis (https://maxunmix.shinyapps.io/MAX_UnMix_final_version/) is useful in evaluating distribution of magnetic coercivity in material (Kruiver et al. 2001; Heslop et al. 2002; Egli 2003).

Because of the existing analytic limit of compositional analysis, stoichiometry of ADS-related dust particles requires confirmation from non-chemical analysis. As an alternative mineral compositional determination, continuous measurement of IRM during zero-field cooling (300–10 K) and warming (10–300 K) was carried out. We produced room temperature saturation isothermal remanent magnetization (SIRM) in an applied field of 1 T, and then, the room temperature SIRM was subjected to cooling in zero field (<0.5 mT) to 10 K and then warming to 300 K, using a Quantum Design MPMS at the Institute of Geology and Geophysics, Chinese Academy of Sciences.

## Results

**Fig. 2:**
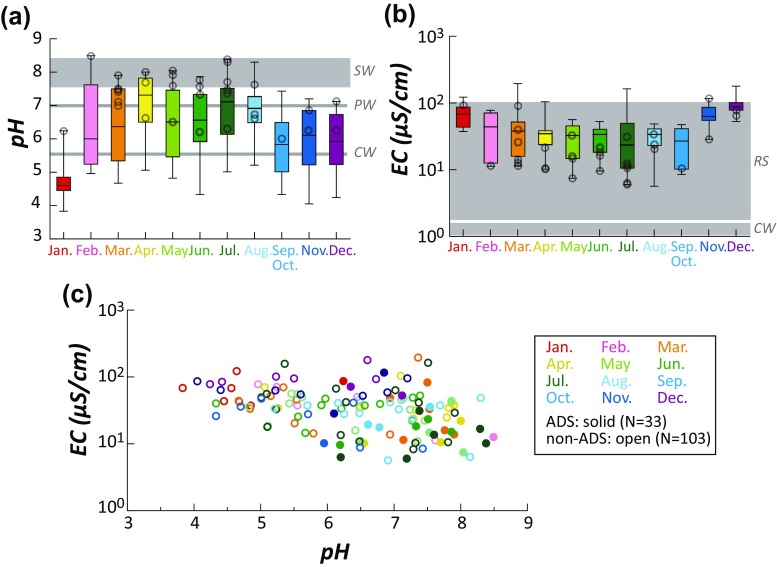
**a** Monthly variations of pH in rainfalls or snow. **b** Monthly variations of EC in rainfalls or snow. **c** Correlation between EC and pH for rainfalls with and without Asian dust storm (ADS) events. *PW* pure water, *SW* seawater, *CW* water in CO_2_ equilibrium with atmosphere, *RS* rain or snow. *Gray circles* denote the occurrence of individual ADS

Monthly variations of pH (Fig. 2a) and EC (Fig. 2b) are displayed in box plots, where central box represents the inter-quartile and whisker lines are extending to include the maximum and minimum. Precipitation acidity is strongly dependent on the presence of acidic and alkaline elements. Pure water (PW) has a neutral pH value of 7, and seawater (SW) typically shows pH from 7.5 to 8.4 (Jenkins 1989). In a clean atmosphere, the pH of precipitated rainwater or snow is expected to be weakly acidic due to dissolution of carbon dioxide (CO_2_) and the existence of background sulfur dioxide (SO_2_) (Börner et al. 2013; Seinfeld and Pandis 2016). If the CO_2_-rich water (CW) is in equilibrium with the atmosphere, the pH would be as low as 5.65 (Börner et al. 2013). In the present study, values of pH were relatively closer to PW over the summer season but were biased towards the value of CW over the winter season (Fig. 2a). In particular, the pH was highly acidic in January (Fig. 2a).

The EC reflects the amount of ionic content in a solution. For instance, pure water is far from being a good conductor with EC < 10^−2^ μS/cm. Typical ranges of EC for CW are 0.2–2.0 μS/cm, while those for rainwater or snow (RS) are 20–100 μS/cm (Atkins 1947; Komabayasi and Isono 1967; Jenkins 1989). In this study, values of EC fell in an envelope of RS (Fig. 2b). It is apparent that values of EC were relatively higher in colder seasons (Fig. 2b), which is opposite the trend observed for pH (Fig. 2a).

Mean values of pH and EC from 33 samples collected during or just after the ADS events (solid symbols in Fig. 2c) were 7.23 ± 0.68 and 19.79 ± 2.12 μS/cm, while those from 103 non-ADS samples (open symbols in Fig. 2c) were 6.14 ± 1.12 and 37.55 ± 2.05 μS/cm, respectively. For comparison, occurrence of individual ADS was displayed as gray circles (Fig. 2a, b). Samples collected during or just after the ADS events tend to have higher pH and lower EC than those irrelevant to ADS events (Fig. 2c).

**Fig. 3:**
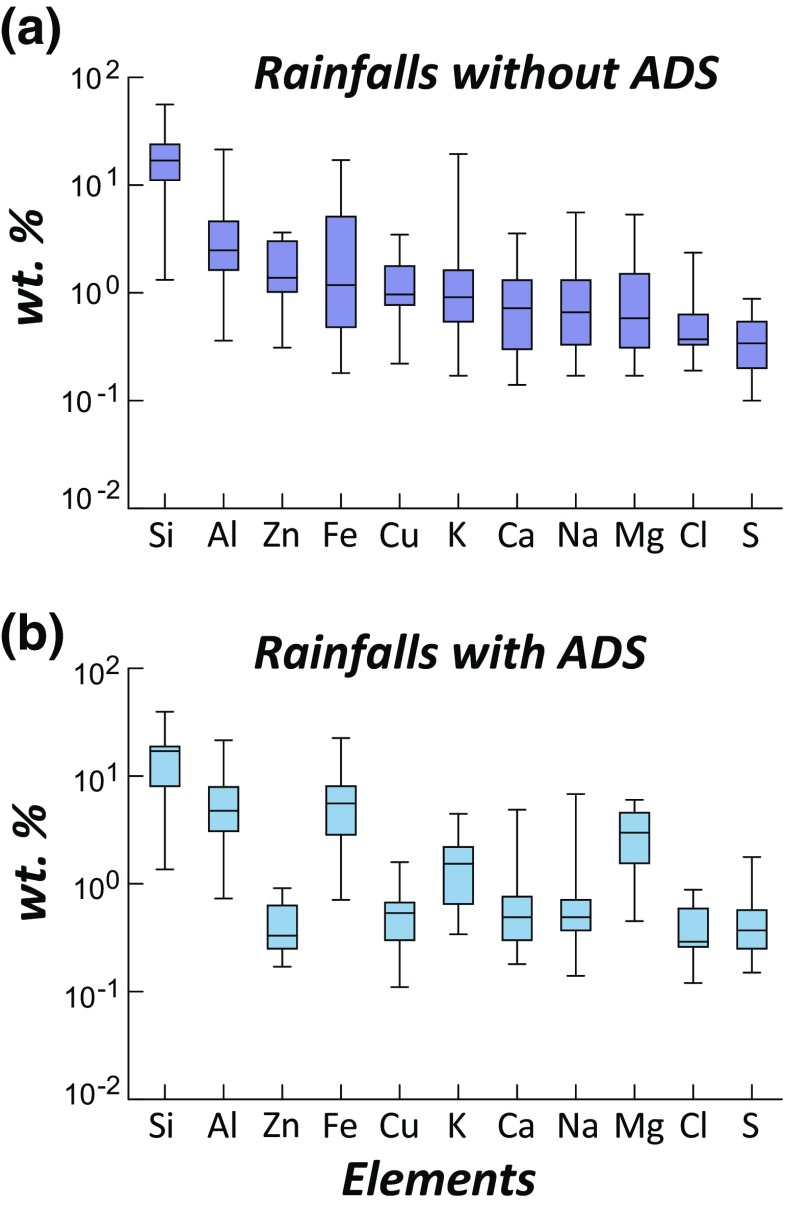
Abundance of elements on rainfalls for **a** non-ADS events and **b** ADS events

Compositional data are also presented in box plots, where central box represents the inter-quartile and whisker lines are extending to include the maximum and minimum (Fig. 3). Solid extracts from non-ADS-related rainwater (Table 1) show the abundance of elements of Si, Al, Zn, Fe, Cu, K, Ca, Na, Mg, Cl, and S in decreasing order (Fig. 3a). Similarly, solid extracts from ADS-related rainwater showed an enrichment of Al, Fe, K, and Mg and a depletion of Zn and Cu (Fig. 3b and Table 1).

**Table 1:** Abundance of elements on rainfalls for non-ADS and ADS events in wt.%

	Without ADS			With ADS		
	Median	Maximum	Minimum	Median	Maximum	Minimum
Si	16.9	55.96	1.32	17.03	39.52	1.36
Al	2.48	21.39	0.36	4.76	21.51	0.73
Zn	1.38	3.63	0.31	0.33	0.91	0.17
Fe	1.18	17.06	0.18	5.58	22.51	0.71
Cu	0.97	3.46	0.22	0.54	1.59	0.11
K	0.91	19.4	0.17	1.54	4.46	0.34
Ca	0.72	3.56	0.14	0.49	4.87	0.18
Na	0.66	5.57	0.17	0.49	6.8	0.14
Mg	0.58	5.33	0.17	2.98	6.02	0.45
Cl	0.37	2.36	0.19	0.29	0.88	0.12
S	0.34	0.88	0.10	0.37	1.77	0.15

**Fig. 4:**
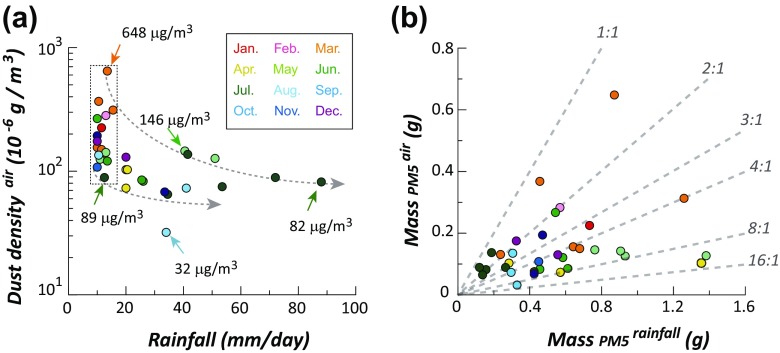
**a** Dust density in air as a function of rainfall. **b** Comparison of PM_5_ mass of mesh-filtered dusts from 33 ADS-related rainfall events with that from the temporal variation of dust density in local stations

Amount of PM_5_ (albeit dust densities) was plotted as a function of rainfall (Fig. 4a). For rainfalls between 10 and 15 mm/day (dashed squares in Fig. 4a), dust density ranges from 89 to 648 μg/m^3^ (Fig. 4a). With increasing rainfalls, dust density plummeted and then remained rather constant with increasing rainfalls (two dashed arrows in Fig. 4a). Such rather constant dust density in air is evident during the summer season (Fig. 4a). There is an outlier with extremely lower dust density of 32 μg/m^3^ recorded on August 11, 2009 (Fig. 4a).

Mass of extracted PM_5_ from 33 ADS-related rainfall events was compared with that predicted from the temporal variation of dust density in local station prior to raining event (Fig. 4b). If raining event is effective in diluting the dust in air, PM_5_ from rainfalls is in relation of 1:1 proportion to that from dust in air (Fig. 4b). Majorities of results are highly biased towards higher concentrations of PM_5_ for rainfall than those of PM_5_ for air. In particular, results obtained from March, April, and May are highly biased towards PM_5_ from rainfalls (Fig. 4b). It is therefore important to emphasize that high rainfall does not guarantee more dilution of dusts in air (Fig. 4b).

**Fig. 5:**
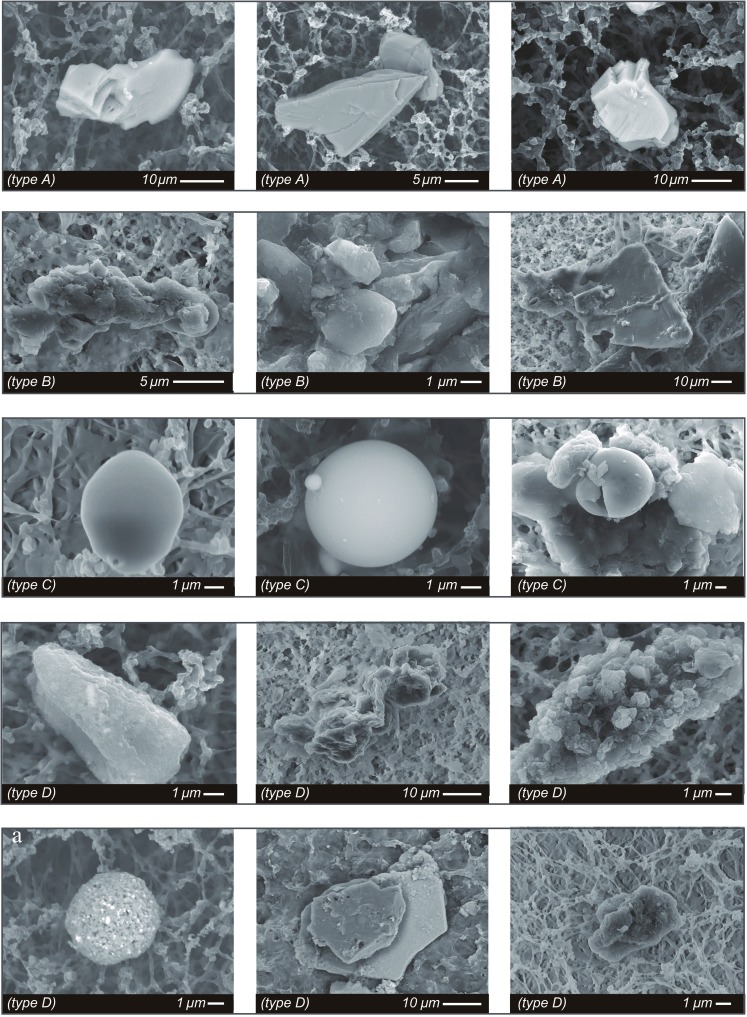
Representative images of dust particles from the mesh-filtered sets of rainwaters

On the basis of shape and compositional analysis, solid particulates were divided into four types in order of abundance. Particles in type A were sharp edged, with average length of 10–40 μm (Fig. 5). They were rich in crustal elements of Si, Al, Fe, K, Na, and Cu. The richest element for type A was Si. On the basis of crystal morphology and elemental composition, it is likely that type A originated from local soil which is basically weathered granite (Fig. 5).

Particles in type B were angular with average diameter of 5–30 μm (Fig. 5). They were composed of mainly Fe and O, with some contribution of crustal elements including Mg, Ca, Al, K, and Na. These angular iron-rich oxide particles were possibly originated from natural processes, such as pedogenesis or weathering (Kim et al. 2007, 2008, 2009).

Particles in type C were mostly rounded or aggregate of ellipsoidal- or mushroom-shaped particulate (Fig. 5). These particles were dominated by Fe and C with minor contributions from Al, Ca, Mg, and Si. In addition, trace amount of Cu, Cr, Zn, and Pb was also common. These rounded Fe-bearing particles were unlikely to represent natural minerals but were produced by fossil fuel combustion process considering their C–Fe contents and morphological characteristics (Matzka and Maher 1999).

Particles in type D were 1–10 μm in length with various morphologies including irregular and aggregate existing together (Fig. 5). They were characterized by their overwhelming carbon contents with less than a few percent contributions of copper, aluminum, and iron; they can be recognized as black carbon of combustion origin (Griffin and Goldberg 1979). It should be highlighted that black carbon has direct effects on climate system by absorbing solar radiation and heating up the atmosphere (Hansen et al. 1997; Jacobson 2001, 2012; Liu et al. 2009; Menon et al. 2002).

**Fig. 6:**
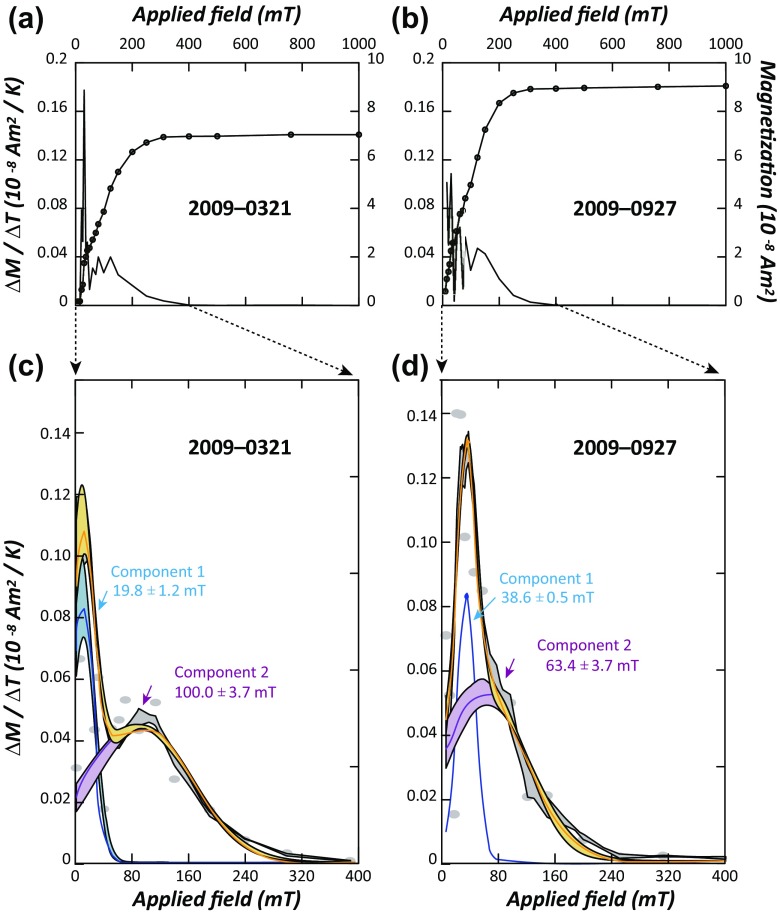
Representative examples of isothermal remanent magnetization (IRM) acquisition. Acquisition of IRM (*circles*) and acquisition rate (Δ*M*/Δ*T*) for **a** 2009-0321 and **b** 2009-0927. IRM component analysis was provided for **c** 2009-0321 and **d** 2009-0927 (https://maxunmix.shinyapps.io/MAX_UnMix_final_version/). **c**, **d** *Blue curves*: the best fit of component 1, *purple curves*: the best fit of component 2, *orange curves*: the fitting sum of two components

During stepwise acquisition of IRM, samples reach saturation near an applied field of 200 mT (Fig. 6a, b). IRM component analysis yielded two magnetic components (Fig. 6c, d). For example, sample 20,090,321 consists of softer component 1 (19.8 ± 1.2 mT) and harder component 2 (100.0 ± 3.7 mT) (Fig. 6c). Similarly, sample 20,090,927 consists of softer component 1 (38.6 ± 0.5 mT) and harder component 2 (63.4 ± 3.7 mT) (Fig. 6d).

**Fig. 7:**
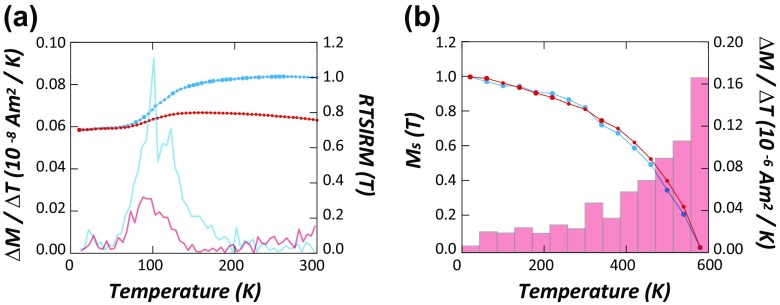
**a** Continuous observation of room temperature saturation isothermal remanence (RT-SIRM) during zero-field cooling from 300 to 10 K (*blue*) and warming from 10 to 300 K (*red*). To induce SIRM, a field of 1 T was applied at 300 K. *Faint lines* represent the variation rates of saturation isothermal remanence. **b** Temperature dependence of saturation magnetization (=*M*_*s*_) at high temperatures indicates the presence of magnetite for type C and type D. *Insets* represent the variation rates of *M*_*s*_ in histograms

During cooling, SIRM decreased prominently from 100 to 120 K, as the rate of remanence change showed two peaks at ∼105 and ∼125 K, respectively (Fig. 7a). During the subsequent warming, the remanence increased more rapidly at the Verwey transition (Verwey 1935), suggesting the presence of magnetite (Özdemir et al. 2002). The presence of magnetic minerals similar to magnetite in ADS is also supported by the temperature dependence of saturation magnetization (Fig. 7b). Both type C and type D PM showed virtually identical temperature dependence of saturation magnetization during heating (Fig. 7b). Observed Curie point both for type C and type D dusts were near 580 °C, again confirming the presence of magnetite (Fig. 7b).

## Discussion

Acid rain poses a serious environmental threat as the pollutants of acid rain can damage human health through the inhalation. The nature of rainwater in Korea appears to be alkaline (spring and summer) to weakly acidic (fall and winter) (Fig. 2a). It is likely that the alkaline nature of rainwater from March to August possibly results from the high precipitation of warmer clouds buffered by soil-derived acidic PM. On the contrary, rainwater becomes more acidic from September to February as the buffering ability of soil dust declines (Fig. 2a). Although rare, the minimum pH of rainwater for each month was substantially lower than that of the CO_2_-equilibrated value of 5.6, indicating that acidic rain deserves serious attention (Fig. 2a). During or just after the ADS events, precipitations were more alkaline (i.e., higher pH) possibly due to abundant supply of alkaline minerals to the precipitation (Kang et al. 2004). It should be noted that the pH level of soils from the deserts source area was 7.9–9.2. As EC represents the ionic abundance but not the composition itself (Fig. 2b), pH is more sensitive in reflecting the chemical variations of rainwater (Fig. 2a). Individual observations of pH and EC showed a weak inverse correlation, although the results are fairly distributed (Fig. 2c). In other words, values of EC were relatively lower in warmer seasons (Fig. 2b), opposite to the trend observed for pH (Fig. 2a).

According to compositional analysis of rainwater with and without ADS, the enrichment of Fe content in ADS-related rainfall events is most eye-catching (Fig. 3 and Table 1). Such enhancement of Fe in ADS-related rainfall events matches well with the magnetic observations. Therefore, it is likely that Fe contents in air dust during ADS events increased. It has been suggested that anthropogenic process induces enhancement of hazardous elements in air, including Fe enrichment from the Fe-smelting plant, Cu enrichment from vehicle traffic, and Pb enrichment from anthropogenic sources (Wei and Yang 2010; Zhang et al. 2012; Lee et al. 2013). Another important observation is that Zn and Cu were efficiently diluted on rainfall events (Fig. 3).

On the basis of dust density variations with rainfall events, it is possible that the wash-out effect of rainfall was most prominent for rainfalls about 10–20 mm/day (Fig. 4a). Of course, it does not necessarily indicate that rainfall over 10–20 mm/day was less effective in diluting dust density in air. Instead, it is more likely that dust was diluted well enough during the early stage of intense rainfalls (Fig. 4b).

Four different types of PM_5_ were recognized in the present study (Fig. 5). They were natural soils (type A), natural dust of pedogenesis or weathering origin (type B), anthropogenic C–Fe-rich particles (type C), and anthropogenic C-rich particles (type D). Sulfides or sulfates can be produced from the oxidation of SO originated from coal-burning and traffic emission and natural sea salt. Despite common documentations on the presence of abundant sulfides in ADS (Streets et al. 2000), magnetic techniques were sensitive in identifying dilution effect of Fe-bearing minerals only because sulfides or sulfates in rainfalls were likely to be non-magnetic (Fig. 6).

It is natural to consider that Fe-rich materials (type C and type D) are anthropogenic in origin. On the basis of HYSPLIT analysis, majorities of ADS passed over the industrialized area in eastern China and western Korea. However, we cannot exclude the possibility that C–Fe-bearing material of local origin of vehicle brake and tire wear might also contribute to the dust signals (Varrica et al. 2013).

Verwey transition (Verwey 1935) and Curie temperature data (Fig. 7) suggest that the main magnetic mineral in ADS dusts is likely to be magnetite. However, compositional data suggest that Fe-rich minerals are far from being stoichiometric magnetite. Considering the carbon-related composition and their unique particle morphology, it is possible that anthropogenic processes might produce individual spherical particles or aggregates of fine-grained magnetite, whose surface is carbon coated as a result of combustion process. If so, presence of double peaks at ∼105 and at ∼125 K on cooling of RT-SIRM can be interpreted as the Verwey transition of magnetite interior (possibly component 1) and that of carbon-coated shell (possible component 2). But then, it is unclear why such contrast disappeared during warming of RT-SIRM (Fig. 7a).

It is intuitively obvious that rainfall dilutes dusts in air. In the present study, we found that Fe-rich material is more effectively concentrated in rainfalls, suggesting that rainfalls are natural pollution filters for Fe-rich material. In the future, magnetic sensitivity can be used as a proxy for anthropogenic contribution of dust storms as a non-destructive and quick scientific technique. In the present study, only water-filtered solid particles of PM_5_ collected from fiber filters were used because the amount of dusts extracted by smaller size filters (PM_3_, PM_1_, PM_0.45_, PM_0.2_, PM_0.1_) were beyond the resolution limits of magnetic instruments available. In the future, larger water tank is necessary to collect more rainfalls to characterize magnetic properties of dusts extracted by smaller-size filters. In addition, it remains technically challengeable to measure magnetic properties of air-filtered dust samples during non-ADS days on short exposure.

## Conclusion

This study demonstrates the following facts:

1. We analyzed a total of 136 rainwaters (or snow during the winter season) collected in Daejeon, Korea, from February 2009 to February 2013. Monthly variations of pH showed that rainfalls in Korea were alkaline from March to August due to high precipitation of warmer clouds buffered by soil-derived particles. On the other hand, rainfalls became weakly acidic in colder seasons as the buffering ability decreased.
2. Monthly variations of EC showed that values of EC were relatively lower in warmer seasons, opposite to the trend observed for pH.
3. Based on the compositional analysis of rainwater with and without ADS, Fe is concentrated in ADS-related rainfall events. Such concentration of Fe in ADS-related rainfall events is supported from magnetic observation. We also found that rainfall events are fairly effective in selectively removing Zn and Cu.
4. Dust densities were denser for rainfalls of 10–15 mm/day. With increasing rainfalls, dust density appears to converge towards 0.1 mg/m^3^.
5. If raining event is effective in diluting the dust in air, dust density with and without rainfalls should be proportional. However, a systematic biased towards higher values of dust density with rainfalls was observed.
6. In the present study, four different types of PM_5_ were recognized as natural soils (type A), natural dust of pedogenesis or weathering origin (type B), anthropogenic C–Fe-rich particles (type C), and anthropogenic C-rich particles (type D).
